# Erratum to: *Education:* The role of the first siderophore cephalosporin Fetcroja^®^ (cefiderocol) in UK clinical practice: introduction; *Education:* An introduction to the core data for cefiderocol with reflections for a possible role within UK clinical practice; *Education:* A compassionate use of cefiderocol to treat osteomyelitis caused by an XDR *Pseudomonas aeruginosa*; Compassionate use of cefiderocol for carbapenem-resistant *Acinetobacter baumannii* prosthetic joint infection; *Education:* An overview from the author of ‘Cefiderocol as rescue therapy for *Acinetobacter baumannii* and other carbapenem-resistant Gram-negative infections in intensive care unit patients’

**DOI:** 10.1093/jacamr/dlab110

**Published:** 2021-08-28

*JAC-Antimicrobial Resistance* 2021; https://doi.org/10.1093/jacamr/dlab051

*JAC-Antimicrobial Resistance* 2021; https://doi.org/10.1093/jacamr/dlab053

*JAC-Antimicrobial Resistance* 2021; https://doi.org/10.1093/jacamr/dlab054

*JAC-Antimicrobial Resistance* 2021; https://doi.org/10.1093/jacamr/dlab055

*JAC-Antimicrobial Resistance* 2021; https://doi.org/10.1093/jacamr/dlab056

In the original published versions of these articles, in the Transparency declarations, “This article forms part of a Supplement sponsored by Shionogi Europe.”, should have read “This article is part of a promotional Supplement developed and sponsored by Shionogi B.V. The accompanying video formed part of an educational webinar for which the author received a speaking honorarium.”

This has now been corrected in the published versions of the articles. The authors apologize for this error.

